# Association between interatrial septum adiposity and atrial fibrillation: transesophageal echocardiography imaging and autopsy study

**DOI:** 10.1038/s41598-023-36677-1

**Published:** 2023-06-17

**Authors:** Miho Miyoshi, Ichitaro Abe, Nozomi Kodama, Yinge Zhan, Shintaro Kira, Yumi Ishii, Taisuke Harada, Masayuki Takano, Masaki Takahashi, Hiroki Sato, Katsunori Tawara, Hidekazu Kondo, Akira Fukui, Tomoko Fukuda, Hidefumi Akioka, Tetsuji Shinohara, Yasushi Teshima, Kunio Yufu, Mikiko Nakagawa, Tsutomu Daa, Tatsuo Shimada, Naohiko Takahashi

**Affiliations:** 1 Department of Cardiology and Clinical Examination, Oita University Faculty of Medicine, 1-1 Idaigaoka, Hasama, Oita, 879-5593 Japan; 2 Medical Education Center, Oita University Faculty of Medicine, Oita, Japan; 3 Department of Diagnostic Pathology, Oita University Faculty of Medicine, Oita, Japan; 4 Oita Medical Technology School, College of Judo Therapy and Acupuncture-Moxibustion, Oita, Japan

**Keywords:** Cardiology, Cardiovascular diseases, Atrial fibrillation

## Abstract

Recent clinical evidence has suggested that interatrial septal (IAS) adiposity contributes to atrial fibrillation (AF). The present study aimed to confirm the usefulness of transesophageal echocardiography (TEE) to estimate IAS adiposity in patients with AF. The histological IAS analysis based on autopsy samples sought to clarify characteristics that underlie the contribution of IAS adiposity to AF. The imaging study analyzed the TEE results in patients with AF (*n* = 184) in comparison with transthoracic echocardiography (TTE) and computed tomography (CT) results. The autopsy study histologically analyzed IAS in subjects with (*n* = 5) and without (*n* = 5) history of AF. In the imaging study, the ratio of interatrial septum adipose tissue (IAS-AT) volume per epicardial adipose tissue (EpAT) volume was greater in patients with persistent AF compared (PerAF) to those with paroxysmal AF (PAF). Multivariable analysis revealed that both TEE-assessed IAS thickness and TTE-assessed left atrial dimension were predicted by CT-assessed IAS-AT volume. In the autopsy study, the histologically-assessed IAS section thickness was greater in the AF group than that in the non-AF group and was positively correlated with the IAS-AT area percentage. In addition, the size of adipocytes in IAS-AT was smaller, compared to EpAT and subcutaneous adipose tissue (SAT). IAS-AT infiltrated into the IAS myocardium, as if adipose tissue split the myocardium (designated as myocardial splitting by IAS-AT). The number of island-like myocardium pieces as a result of myocardial splitting by IAS-AT was greater in the AF group than in the non-AF group and was positively correlated with the IAS-AT area percentage. The present imaging study confirmed the usefulness of TEE to estimate IAS adiposity in patients with AF without radiation exposure. The autopsy study suggested that the myocardial splitting by IAS-AT may contribute to atrial cardiomyopathy leading to AF.

## Introduction

A growing body of evidence has shown that epicardial adipose tissue (EpAT) is associated with atrial fibrillation (AF)^1–5^. In fact, using human left atrial appendage samples, we have recently demonstrated that fibrotic remodelling of EpAT and cytokines/chemokines, including interleukin-6, monocyte chemoattractant protein-1, and tumor necrosis factor-α, had crucial roles in atrial myocardial fibrosis^3^. In addition, Venteclef et al.^4^ and our group^5^ have utilized an organo-culture system to experimentally demonstrate that paracrine effects of human EpAT cause severe structural remodelling in rat atrial myocardium^4,5^. Recently, EpAT has been reportedly associated with atrial conduction time^6^. In this regard, obesity has been associated with atrial conduction slowing and greater electrogram fractionation^7^.

**Figure 1:**
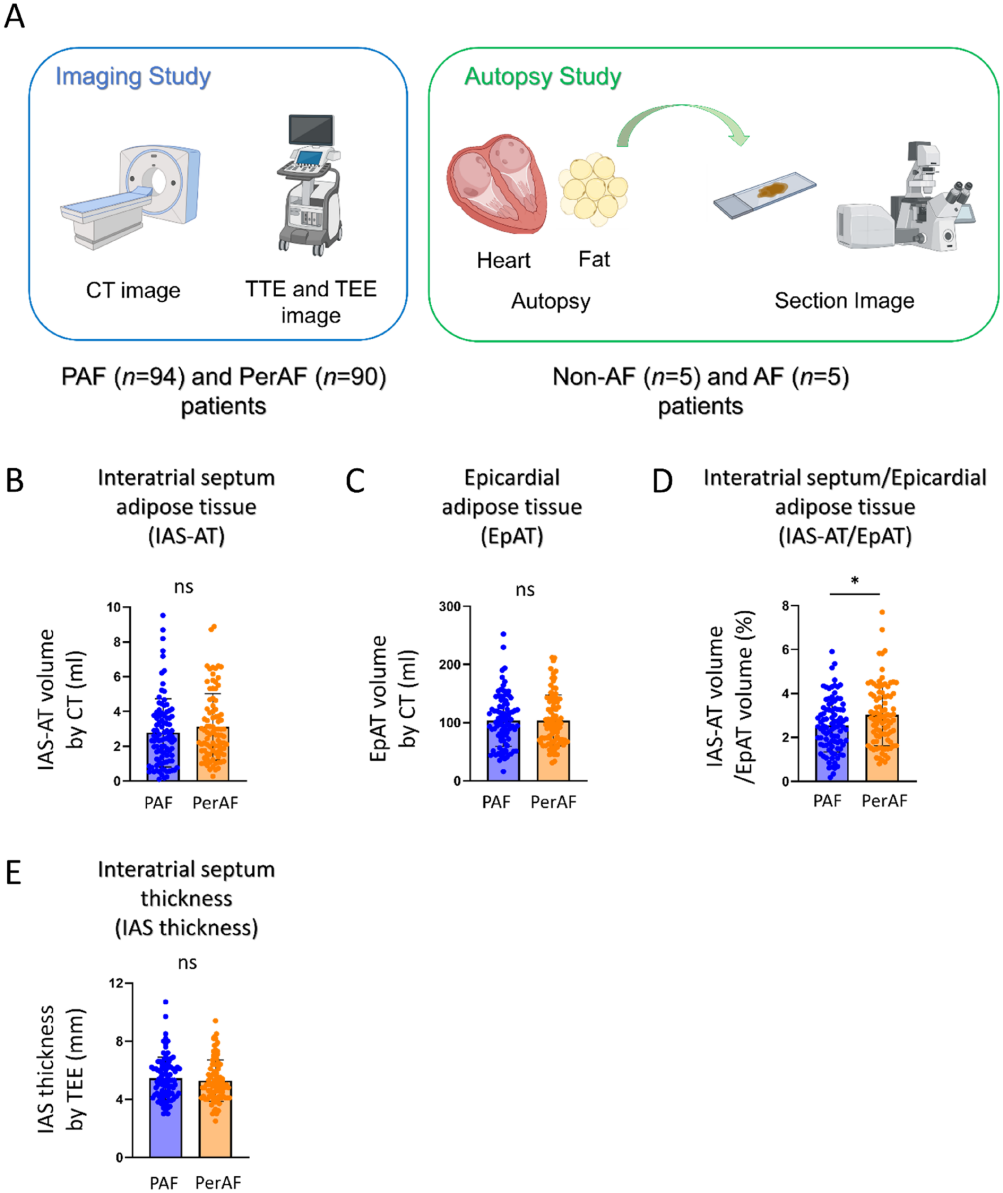
Schematic illustration of the study population and comparison of interatrial septum adipose tissue (IAS-AT) volume among patients with paroxysmal AF (PAF) and those with persistent AF (PerAF). (**A**) Schematic illustration of the study population for the imaging study in PAF (*n* = 94) and PerAF (*n* = 90) patients, and for the autopsy study in Non-AF (*n* = 5) and AF (*n* = 5) patients. (**B**) Interatrial septum adipose tissue (IAS-AT) volume assessed by CT. Data are presented as means ± SD. ns, not significant by Student’s *t*-test. *n* = 94 for PAF, *n* = 90 for PerAF. (**C**) Epicardial adipose tissue (EpAT) volume assessed by CT. Data are presented as means ± SD. ns, not significant by Student’s *t*-test. *n* = 94 for PAF, *n* = 90 for PerAF. (**D**) IAS-AT volume/EpAT volume assessed by CT. Data are presented as means ± SD. *p < 0.05 by Student’s *t*-test. *n* = 94 for PAF, *n* = 90 for PerAF. (**E**) Interatrial septum (IAS) thickness assessed by transesophageal echocardiography (TEE). Data are presented as the means ± SD. ns, not significant by Student’s *t*-test. *n* = 94 for PAF, *n* = 90 for PerAF.

The interatrial septum (IAS) has been traditionally regarded as a fibromuscular structure separating the right atrium (RA) and left atrium (LA), which is an anatomic extension of EpAT^8,9^. Electrophysiologically, IAS plays an important role in both interatrial and intra-atrial conduction^10,11^. It has been demonstrated that IAS thickness was increased in patients with AF^12,13^, and that an increased IAS thickness was associated with frequent recurrence after AF catheter ablation^14^. The thickened IAS has been associated with abundant IAS adiposity^15^. In this regard, Samanta et al.^16^ have recently shown that the interatrial septum adipose tissue (IAS-AT) volume is associated with prolonged transseptal conduction time and P-wave duration. However, how IAS adiposity is associated with atrial conduction disturbance and AF has not been fully elucidated. To clarify this, we first attempted to confirm the usefulness of transesophageal echocardiography (TEE) to evaluate IAS adiposity in patients with AF. The histological IAS results from autopsies were analyzed to determine characteristics that underlie the contribution of IAS adiposity to AF (Fig. 1A).

## Results

### Imaging study

#### Patient characteristics

Clinical characteristics of the enrolled AF patients are summarized in Table 1. The mean age was 68.7 ± 8.9 years, and 28% of patients were female. The mean CHADS_2_ score was 1.6 ± 1.2. A total of 61% of patients had a history of hypertension and 22% of patients were diabetic. Ninety-four patients had paroxysmal AF (PAF), whereas ninety patients had persistent AF (PerAF). The left atrial diameter (LAD) measured by transthoracic echocardiography (TTE) was larger in patients with PerAF compared to those with PAF (43.1 ± 4.6 vs*.* 38.8 ± 5.7 mm, *p* < 0.01). Left ventricular ejection fraction (LVEF) was significantly different between the PAF and PerAF groups (65.1 ± 10.5 vs. 60.2 ± 12.0%, *p* < 0.01).

**Table 1:** Patient characteristics of 184 patients.

	All patients (*n* = 184)	Paroxysmal AF (*n* = 94)	Persistent AF (*n* = 90)
Age (years)	68.7 ± 8.9	70.1 ± 7.6	67.2 ± 9.9*
Sex			
Male, n (%)	133 (72)	60 (64)	73 (81)**
Female, n (%)	51 (27)	34 (36)	17 (19)**
BMI (kg/m^2^)	24.5 ± 3.1	24.0 ± 2.9	24.9 ± 3.1*
CHADS_2_ score	1.6 ± 1.2	1.76 ± 1.14	1.50 ± 1.16
HT, n (%)	113 (61)	66 (70)	47 (52)*
DM, n (%)	41 (22)	20 (21)	21 (23)
HF, n (%)	36 (20)	11 (12)	25 (28)**
Medication			
ACEI or ARB, n (%)	77 (42)	39 (41)	38 (42)
* β*-blocker, n (%)	85 (46)	41 (44)	44 (49)
Statin, n (%)	64 (35)	34 (36)	30 (33)
Amiodarone, n (%)	52 (28)	16 (17)	36 (40)**
Labo data			
Hb (g/dl)	13.8 ± 1.7	13.4 ± 1.5	14.2 ± 1.89**
Cr (mg/dl)	1.1 ± 1.2	1.14 ± 1.44	1.04 ± 0.82
eGFR (ml/min/1.73 m^2^)	61.7 ± 19.1	62.9 ± 21.0	60.6 ± 16.7
pro BNP (pg/ml)	918.8 ± 2917.0	414.8 ± 689.7	1434.3 ± 4024.9*
HbA1c (%)	5.9 ± 0.6	5.9 ± 0.6	6.0 ± 0.6
LDL-C (mg/dl)	106.5 ± 31.3	109.1 ± 29.1	103.8 ± 33.2
HDL-C (mg/dl)	57.3 ± 14.4	58.1 ± 14.1	56.4 ± 14.7
TG (mg/dl)	116.3 ± 68.9	113.3 ± 65.2	119.5 ± 72.5
Echo data			
LAD (mm)	40.9 ± 5.6	38.8 ± 5.7	43.1 ± 4.6**
LVDd (mm)	48.5 ± 5.5	47.7 ± 5.3	49.3 ± 5.6
LVDs (mm)	31.9 ± 6.3	30.7 ± 6.1	33.3 ± 6.3**
LVEF (%)	62.7 ± 11.6	65.1 ± 10.5	60.2 ± 12.0**
E/e′	12.7 ± 5.3	12.9 ± 5.4	12.5 ± 5.1

Data are given as mean ± SD or n (%). *p < 0.05, **p < 0.01, vs PAF.

*AF* atrial fibrillation, *BMI* body mass index, *HT* hypertension, *DM* diabetes mellitus, *HF* heart failure, *ACEI/ARB* angiotensin converting enzyme inhibitors/angiotensin II receptor blocker, *Hb* hemoglobin, *Cr* creatinine, *eGFR* estimated glomerular filtration rate, *pro BNP* pro brain natriuretic peptide, *HbA1c* glycated hemoglobin, *LDL-C* low density lipoprotein cholesterol, *HDL-C* high density lipoprotein cholesterol, *TG* triglyceride, *LAD* left atrial diameter, *LVDd* left ventricular end-diastolic diameter, *LVDs* left ventricular end-systolic diameter, *LVEF* left ventricular ejection fraction.

#### IAS-AT volume assessed by computed tomography (CT) and clinical variables

The IAS-AT volume assessed by CT was positively correlated with body mass index (BMI), CT-assessed EpAT volume, and TTE-assessed LAD (*p* < 0.01 for all; Supplemental Tables S1 and S2).

#### Comparison of CT-assessed IAS-AT volume among patients with PAF and those with PerAF

The IAS-AT volume assessed by CT tended to be greater in patients with PerAF compared to those with PAF, although this difference did not reach statistical significance (2.78 ± 1.96 vs. 3.12 ± 1.96 mL, *p* = 0.23, Fig. 1B). There were no differences in EpAT volume assessed by CT among patients with PAF compared to those with PerAF (103.7 ± 44.3 vs. 103.7 ± 43.8 mL, Fig. 1C). However, the ratio of IAS-AT volume to EpAT volume was significantly greater in patients with PerAF compared to those with PAF (2.54 ± 1.20 vs. 3.01 ± 1.39%, *p* < 0.05, Fig. 1D).

#### TEE-assessed IAS thickness and clinical variables

The TEE-assessed IAS thickness was positively correlated with BMI, CT-assessed EpAT volume, and CT-assessed IAS-AT volume (*p* < 0.01 for all; Supplemental Tables S3 and S4).

#### Comparison of TEE-assessed IAS thickness among patients with PAF and those with PerAF

There was no significant difference in the TEE- assessed IAS thickness among patients with PAF and those with PerAF (PAF 5.44 ± 1.45 vs. PerAF 5.28 ± 1.42 mm, *p* = ns; Fig. 1E).

#### Predictive value for TEE-assessed IAS thickness and TTE-assessed LAD

The univariate and multivariate analyses predicted the TEE-assessed IAS thickness (Table 2). The multivariable analysis revealed that the TEE-assessed IAS thickness was predicted by both CT-assessed IAS-AT volume and BMI (*p* < 0.0001 and *p* = 0.0271, respectively). The univariate and multivariate analyses predicted the TTE-assessed LAD (Table 3). The multivariable analysis revealed that the TTE-assessed LAD was predicted by the CT-assessed IAS-AT volume (*p* = 0.0173).

**Table 2:** Multivariate regression analysis to predict IAS thickness.

	Univariate analysis	Multivariate analysis								
			Model 1		Model 2		Model 3		Model 4	
		Corrected R^2^	0.2762		0.3088		0.3094		0.3206	
		*p* value	< 0.0001		< 0.0001		< 0.0001		< 0.0001	
		Parameter	Estimate	*p* value	Estimate	*p* value	Estimate	*p* value	Estimate	*p* value
IAS-AT volume	< 0.0001		0.3908	< 0.0001	0.3201	< 0.0001	0.3144	< 0.0001	0.3254	< 0.0001
Age	0.5599		0.0167	0.1040	0.0218	0.0353	0.0201	0.1046	0.0197	0.1137
Gender (female)	0.0109				− 0.1822	0.0777	− 0.1887	0.0710	− 0.2048	0.0552
BMI	< 0.0001				0.0749	0.0261	0.0800	0.0227	0.0836	0.0271
CHADS_2_ score	0.2108						− 0.0034	0.9710	0.0211	0.8242
eGFR	0.4378						− 0.0041	0.4162	− 0.0053	0.2917
LVEF	0.5798								0.0083	0.3276
LAD	0.1009								− 0.0175	0.3290

*IAS-AT* interatrial septum adipose tissue, *BMI* body mass index, *eGFR* estimated glomerular filtration rate, *LVEF* left ventricular ejection fraction, *LAD* left atrial diameter.

**Table 3:** Multivariate regression analysis to predict LAD.

	Univariate analysis	Multivariate analysis								
			Model 1		Model 2		Model 3		Model 4	
		Corrected R^2^	0.0801		0.0836		0.0855		0.1088	
		*p* value	0.0006		0.0037		0.0073		0.0024	
		Parameter	Estimate	*p* value	Estimate	*p* value	Estimate	*p* value	Estimate	*p* value
IAS-AT volume	0.0001		0.7151	0.0103	0.7578	0.0089	0.7509	0.0097	0.6867	0.0173
EpAT volume	0.0038		0.0065	0.5912	0.0050	0.6854	0.00509	0.6830	0.0087	0.4863
Age	0.6720				0.0382	0.4147	0.0216	0.6916	0.0232	0.6675
Gender (female)	0.6512				− 0.0004	0.9993	0.0199	0.9662	0.1876	0.6904
CHADS_2_ score	0.3650						0.2487	0.5486	0.1408	0.7335
LVEF	0.0278								− 0.0769	0.0340

*LAD* left atrial diameter, *IAS-AT* interatrial septum adipose tissue, *EpAT* epicardial adipose tissue, *LVEF* left ventricular ejection fraction.

#### Imaging study in non-AF patients

To solve the lack of a non-AF group, we have analyzed 19 non-AF patients who underwent TEE (Supplemental Table S5). The mean age was 54.1 ± 22.9 years, and 63% of patients were female. The mean LAD measured by TEE was 34.7 ± 6.3 mm, and LVEF was 66.2 ± 5.6%. Compared to AF patients (*n* = 184, Table 1), the IAS-AT volume assessed by CT, EpAT volume assessed by CT, and TEE-assessed IAS thickness were significantly less in non-AF patients (*p* < 0.001, *p* < 0.01, and *p* < 0.05, respectively, Supplemental Fig. S1).

### Autopsy study

#### IAS adiposity and IAS thickness

**Figure 2:**
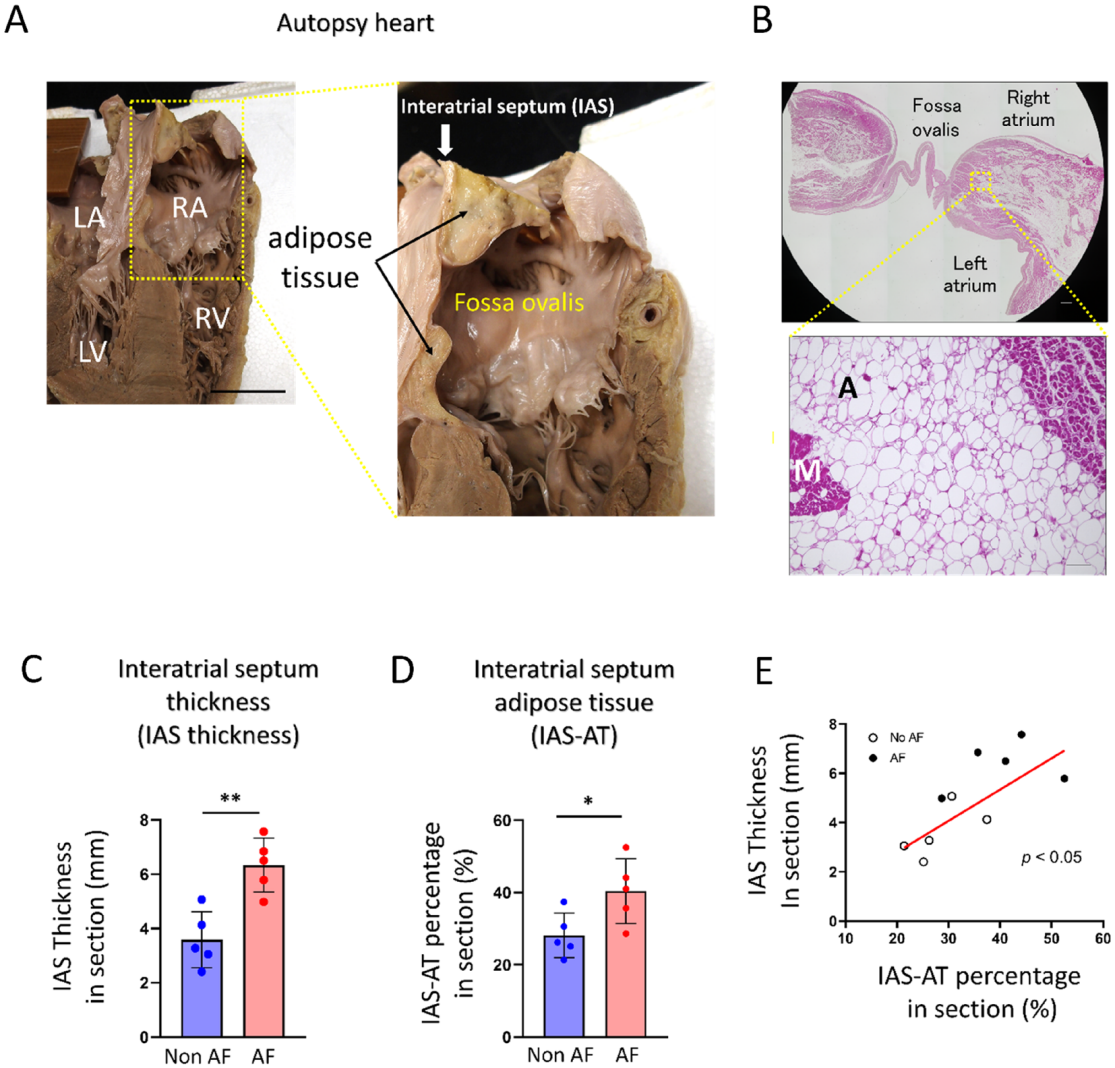
Infiltration of adipose tissue into atrial myocardium in interatrial septum myocardium. (**A**) Representative (left panel) and magnified (right panel) images of human autopsied hearts. LA, left atrium; RA, right atrium; LV, left ventricle; RV, right ventricle. Scale bar: 5 cm. (**B**) Representative (upper panel) and magnified (lower panel) images of interatrial septum (IAS) section. Scale bar: 1 mm (upper), 100 µm (lower). (**C**) Quantitative analysis of histologically-assessed IAS thickness in IAS section. Data are presented as the means ± SD. **p < 0.01, by Student’s *t*-test. *n* = 5 for Non AF, *n* = 5 for AF. (**D**) Quantitative analysis of interatrial septum adipose tissue (IAS-AT) percentage in section. Data are presented as the means ± SD. *p < 0.05, by Student’s *t*-test. *n* = 5 for Non AF, *n* = 5 for AF. (**E**) Correlation between IAS thickness and IAS-AT percentage, by bivariate analysis.* n* = 10 for the group.

Figure 2A shows the representative macroscopic findings of the autopsied heart (Supplemental Table S6). In this case, the excised IAS samples consisted of abundant adipose tissue, which was predominantly located on the right side of the IAS (black arrows, right panel of Fig. 2A). Figure 2B demonstrates the corresponding microscopic hematoxylin–eosin (HE) staining. Histologically-assessed IAS thickness was 6.9 mm. As shown, adipose tissue abundantly infiltrated into the atrial myocardium. Quantitative analyses revealed that the histologically-assessed IAS thickness was significantly thicker in the AF group than in the non-AF group (6.3 ± 0.9 mm vs. 3.6 ± 1.0 mm, *p* < 0.01, Fig. 2C). Similarly, the IAS-AT area percentage in the section was significantly greater in the AF group than that in the non-AF group (40.4 ± 8.9% vs. 28.2 ± 6.1%, *p* < 0.05, Fig. 2D). As a result, the histologically-assessed IAS thickness was positively correlated with the area percentage of IAS-AT (r = 0.5476, *p* < 0.05; Fig. 2E).

#### Histological differences among IAS-AT, EpAT, and subcutaneous adipose tissue (SAT)

**Figure 3:**
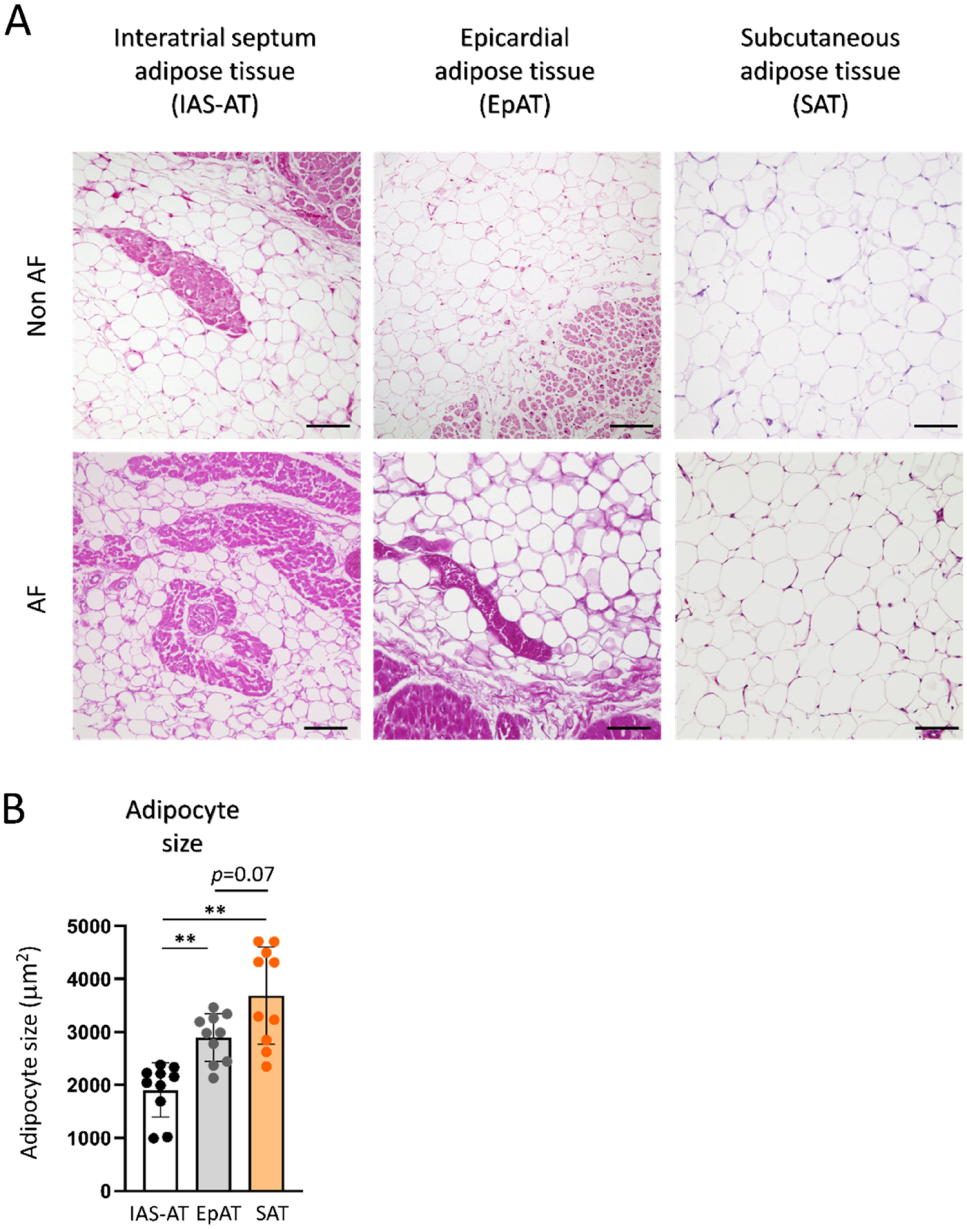
Histological difference among interatrial septum adipose tissue (IAS-AT), epicardial adipose tissue (EpAT), and subcutaneous adipose tissue (SAT). (**A**) Representative images of interatrial septum adipose tissue (IAS-AT), epicardial adipose tissue (EpAT) and subcutaneous adipose tissue (SAT) in Non AF group and AF group. Scale bar: 100 µm. (**B**) Quantitative analysis of adipocyte size. Data are presented as the means ± SD. *p < 0.05, **p < 0.01, by Welch’s correction. *n* = 10 for each group.

Figure 3A shows the HE staining that demonstrates the IAS-AT, EpAT, and SAT from Non AF and AF patients. It was clear that the size of adipocytes in the IAS-AT was smaller than that in the EpAT and SAT. Compared to the EpAT (2895 ± 452 µm^2^) and SAT (3689 ± 915 µm^2^), IAS-AT consisted of smaller-sized adipocytes (1905 ± 511 µm^2^, *p* < 0.01 for both, respectively; Fig. 3B and Supplemental Fig. S2).

#### Myocardial splitting by IAS-AT

**Figure 4:**
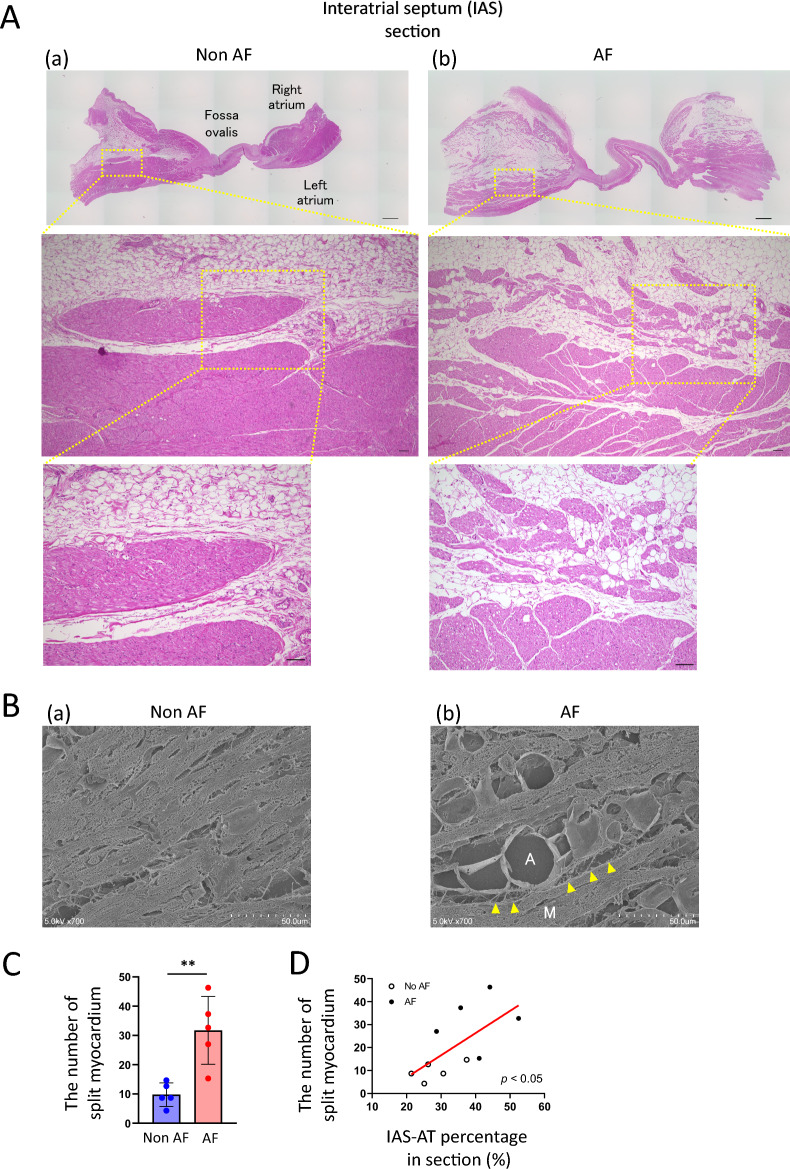
Myocardial splitting by interatrial septum adipose tissue (IAS-AT). (**A**) Representative and magnified images of interatrial septum (IAS) sections in a patient without (a) and with (b) AF. Scale bar: 1 mm (upper), 100 µm (middle), 100 µm (bottom). (**B**) Representative SEM images in patients without (a) or with (b) AF. A, adipocyte; M, myocardium. Yellow arrowheads indicate collagen fibers neighboring adipocytes. (**C**) Quantitative analysis of the number of splitting myocardium pieces. Data are presented as the means ± SD. **p < 0.01, by Student’s *t*-test. *n* = 5 for Non AF, *n* = 5 for AF. (**D**) Positive correlation between interatrial septum adipose tissue (IAS-AT) percentage in sections and number of split myocardium pieces, by bivariate analysis.* n* = 10 for the group.

Supplemental Fig. S3 shows the IAS obtained from AF group. As shown in the right upper panel, the IAS-AT infiltrated into the IAS myocardium, as if adipose tissue split the myocardium. This characteristic finding was designated as “myocardial splitting by IAS-AT”. A greater magnification (right lower panel) demonstrated the infiltration of heterogeneously-sized adipocytes into the atrial myocardium. Figure 4A shows the two representative HE-stained IAS sections in a patient without AF and with AF. In the non-AF case, the adipocyte infiltration was lower, and the myocardial splitting by IAS-AT was scarcely observed (Fig. 4A(a)). In contrast, adipocytes abundantly infiltrated into the atrial myocardium in the AF group case, revealing the myocardial splitting by IAS-AT (Fig. 4A(b)). Moreover, Fig. 4B demonstrates the representative scanning electron microscopy (SEM) images in a patient without AF and with AF. As shown on Fig. 4B(a), the adipocyte infiltration and the amount of surrounding collagen were lower in the non AF group patient. In contrast, the interstitial adipocytes and neighboring collagen fibers (yellow allows) were abundantly observed in the patient with AF group patient (Fig. 4B(b)).

As shown in Fig. 4A, the myocardial splitting by IAS-AT was observed in all 10 autopsies to a varied extent. Three random images, including the border between atrial myocardium and IAS-AT, at 40× magnification per section were analyzed in order to manually count the number of island-like myocardium fragments in each of 10 autopsies to obtain the mean values. As a result of the myocardial splitting by IAS-AT, the number of island-like myocardium pieces was significantly greater in the AF group than in the non-AF group (31.7 ± 11.6 vs. 9.8 ± 4.0 pieces, *p* < 0.05, Fig. 4C). This number per section was positively correlated with the IAS-AT section area percentage (r = 0.6612, *p* < 0.05; Fig. 4D).

#### Left atrial EpAT

The representative LA section images in a patient without AF and patient with AF are shown in Supplemental Fig. S4A. In all 10 autopsies, the EpAT area percentage in the LA section was significantly greater in the AF group than that in the non-AF group (41.6 ± 2.0% vs. 24.4 ± 13.4%, *p* < 0.05, Supplemental Fig. S4B).

## Discussion

### Major findings

The present study uncovered several major findings.

In the imaging study:

1. The ratio of IAS-AT volume per EpAT volume assessed by CT was significantly greater in patients with PerAF compared to those with PAF.
2. The multivariable analysis revealed that both TEE-assessed IAS thickness and TTE-assessed LAD were predicted by CT-assessed IAS-AT volume.

In the autopsy study:

1. The histologically-assessed IAS section thickness was greater in the AF group than that in the non-AF group and was positively correlated with the IAS-AT area percentage.
2. Compared to EpAT and SAT, IAS-AT consisted of adipocytes of smaller size.
3. The IAS-AT infiltrated into the IAS myocardium, as if adipose tissue split the myocardium (myocardial splitting by IAS-AT).
4. The number of island-like myocardium pieces was significantly greater in the AF group than that in the non-AF group as a result of myocardial splitting by IAS-AT and was positively correlated with the IAS-AT area percentage.

Because the number of subjects was small and their background was heterogeneous in the present autopsy study, it is difficult to reach a conclusion when comparing the findings between the AF and non-AF groups. Nevertheless, the myocardial splitting by IAS-AT was clearly associated with IAS adiposity. Therefore, the myocardial splitting by IAS-AT may contribute, at least in part, to the atrial cardiomyopathy leading to AF, especially in overweight subjects.

### Clinical significance for EpAT, IAS-AT volume, and IAS thickness evaluation using imaging modalities

The CT results showing that the ratio of IAS-AT to EpAT volume was significantly greater in patients with PerAF compared to those with PAF suggest that IAS adiposity may be more profoundly associated with AF progression compared to EpAT adiposity. On the other hand, although TEE-assessed IAS thickness was not different among patients with PAF and those with PerAF, it was positively correlated with BMI, CT-assessed EpAT, and CT-assessed IAS-AT volume. Therefore, the assessment of IAS thickness by TEE is useful for estimating the IAS adiposity in individual AF patients without radiation exposure.

Furthermore, the multivariable analysis revealed that TTE-assessed LAD was predicted by CT-assessed IAS-AT volume, suggesting that IAS adiposity may contribute to atrial cardiomyopathy.

### Difference in adipocyte size among IAS-AT, EpAT, and SAT

The finding showed that IAS-AT was composed of smaller-sized adipocytes compared to EpAT and SAT is novel. The adipose tissue mass expansion is characterized by the increase in adipocyte size and/or number^17^. It has been demonstrated that mechanical stress and inflammation cause changes in preadipocyte differentiation^18,19^. Antonopoulos et al.^20^ demonstrated the phenotypic differences between peri-coronary EpAT and SAT, which included adipocyte size and mRNA expression of adipogenesis-related genes. In this regard, we have recently demonstrated that the adipocyte size was smaller, the mRNA expression of inflammation- and fibrosis-related genes was upregulated, and adipogenesis-related genes were downregulated in adipocytes adjacent to the atrial myocardium compared to those located further away^21^. Taken together, these data suggest that the IAS-AT, which is routinely exposed to dynamic mechanical forces between the LA (high pressure) and RA (low pressure), might have different quality and greater inflammatory state compared to EpAT, resulting in smaller size of adipocyte.

### EpAT and IAS-AT in atrial cardiomyopathy

A number of studies have demonstrated the involvement of EpAT in AF^1,2,22,23^. An abundance of EpAT has been reportedly associated with direct adipocyte infiltration into the adjacent atrial myocardium^24,25^. Direct infiltration of adipocytes may also result in conduction slowing^26,27^. In fact, EpAT was independently associated with atrial conduction time in a large-population study as indicated by P-wave indices^6^. Mahajan R et al.^7^ divided 26 patients with AF who underwent AF ablation into two groups at the cut-off point of BMI = 27 kg/m^2^ (obese and reference) and demonstrated that obesity was associated with electroanatomical remodelling of the atria, with areas of low voltage, conduction slowing, and greater fractionation of electrograms. Interestingly, those changes were more pronounced in regions adjacent to the epicardial fat depots. Recently, Nalliah et al.^28^ recruited patients without AF undergoing coronary artery bypass surgery and demonstrated that a greater local EpAT volume was clinically correlated with slower conduction, greater electrogram fractionation, increased fibrosis, and lateralization of cardiomyocyte connexin-40. Although IAS reportedly plays an important role in both interatrial and intra-atrial conduction^10,11^, there has been no direct evidence demonstrating the association between IAS adiposity and conduction disturbance. In this regard, Samanta et al.^16^ recently showed that the IAS-AT volume is associated with prolonged transseptal conduction time and P-wave duration. It is of note that they concluded that these characteristics may increase AF vulnerability in obese patients and heighten the risk of AF recurrence after pulmonary vein isolation^16^. Myocardial splitting by IAS-AT, which is the novel result in the present study, was specifically observed in IAS. In addition, the number of island-like myocardium pieces was positively correlated with the IAS-AT area percentage as a result of myocardial splitting by IAS-AT. Considering these results together with the observations by Samanta et al.^16^, it is strongly suggested that myocardial splitting by IAS-AT, which is associated with IAS adiposity, may underlie the contribution of IAS adiposity to AF. This hypothesis may explain, at least in part, the high recurrence of AF following pulmonary vein isolation in overweight subjects observed in previous research studies^14,16^.

## Limitations

There are some limitations in the present study. Due to the inability to obtain TEE data from age and sex-matched patients, a direct comparison could not be made between groups with matched age and gender. This study was neither prospective nor experimental, but was an observational study. The sample size of this study was small, and it was not possible to exclude the potential contribution of left ventricular functions. Further prospective studies are needed to elucidate the details of our findings. And also, to validate the usefulness of the TEE-assessed IAS thickness, direct comparisons between CT and TEE are needed in the future experiment.

## Conclusion

The present imaging study confirmed the usefulness of TEE to evaluate IAS adiposity in individual AF patients without radiation exposure. The autopsy study suggested that the myocardial splitting by IAS-AT may contribute to the atrial cardiomyopathy leading to AF.

## Methods

Materials and methods are described in detail in the Supplemental materials.

### Imaging study populations

A total of 184 consecutive patients (51 females; age: 68.7 ± 8.9 years) with AF who underwent AF catheter ablation between September 2017 and August 2018 were enrolled in the study. The AF burden was classified according to the American Heart Association/American College of Cardiology/Heart Rhythm Society guidelines^29^. 19 non-AF patients (12 females; age: 54.1 ± 22.9 years) who underwent TEE for the evaluation of the severity of valvular heart diseases and infective endocarditis were also enrolled. All patients were evaluated via blood sampling tests, TTE, TEE, and chest CT. The study protocol was approved by the Ethics Committee of Oita University Hospital (approval number: 1283). The present study was conducted in accordance with the guidelines proposed in the Declaration of Helsinki.

### Quantification of EpAT and IAT volume assessed by CT imaging

The EpAT and IAS-AT volumes were quantified semi-automatically using a high-speed three-dimensional image analysis system (Synapse Vincent; Fuji Photo Film, Tokyo, Japan) on CT images. To analyse the EpAT volume, the pericardium was traced manually from the right pulmonary artery to the diaphragm to determine the region of interest (ROI). Within the ROI, the adipose tissue was defined as pixels within a window of − 195 to − 45 hounsfield units (HU). Overall, only pixels with an HU equivalent to adipose tissue within the pericardial sac were considered as EpAT, as previously described^21^. To analyse the IAS-AT volume, the IAS was traced manually from the top of left atria to the bottom of left atria, which is surrounded by the right atria, left atria, and aorta, to determine the ROI. Within the ROI, adipose tissue was defined as pixels within a window between − 195 and − 45 HU. Only pixels with an HU equivalent to adipose tissue within the IAS were considered as IAS-AT (Supplemental Fig. S5).

### IAS thickness assessed by TEE

TEE was performed after overnight fasting. Lidocaine was used for topical anesthesia of the hypopharynx, and conscious sedation was achieved with midazolam combined with fentanyl or meperidine. With the patient in the partial left lateral position, a multiplane 9-mm TEE probe tipped with a 5.0-MHz miniaturized phased-array ultrasound transducer (Vivid E9, GE Vingmed, Horten, Norway) was inserted into the esophagus and advanced to perform the test. Image depth and sector width were set to maximize the frame rate and the velocity scale was set accordingly to maximize color flow valvular regurgitation analysis while limiting the aliasing effect. The bicaval view was used (transverse midesophageal short-axis view at the level of the aortic valve with the transducer plane at 90–110°) to obtain maximal measurements of the interatrial septum in end-systole 1 cm superior to the fossa ovalis (Supplemental Fig. S6). The depth was adjusted so that all measurements were performed on the same scale in all experiments. We adjusted the TEE projection from which the septal measurements were taken perpendicular to the septum.

### Autopsy study population

Human IAS and LA samples were obtained from autopsies of subjects with (AF group, n = 5) and without AF (non-AF group, n = 5) history and were used for histological analysis. The two groups were age- and sex-matched. Clinical characteristics of 10 autopsies are summarized in Supplemental Table S6. The study protocol was approved by the Ethics Committee of Oita University Hospital (approval number: 1009). Written informed consent was obtained on behalf of each patient prior to the inclusion, as previously described^30^. The study was conducted in accordance with the guidelines proposed in the Declaration of Helsinki. The present study is registered at the University Hospital Medical Information Network (UMIN) Clinical Trials Registry (UMIN000026153). The age, gender, and BMI in the non AF and AF groups are demonstrated in Supplemental Table S7.

### Histological study of autopsy heart tissue

The IAS and LA samples from 10 autopsies were fixed in 4% paraformaldehyde, embedded in paraffin, and cut into 5-μm sections for histological analysis. After deparaffinization, serial sections were stained with HE. Images were acquired and digitized on a BIOLEVO BZ-9000 epifluorescence microscope (Keyence, Osaka, Japan). Adipose tissue from all sections was quantified using semi-automated digital processing (Keyence). The IAS section thickness was determined at a distance 3 mm away from the fossa ovalis. To validate the visually assessed adipocyte size, three random images from each of all 10 autopsies at 100× magnification per section were analyzed to obtain the mean values. Percentage of adipose tissue was determined by calculating the ratio of adipose tissue area to total tissue area. To validate the visually assessed severity of adipocyte infiltration into endocardium, three random images from each of all 10 autopsies at 40× magnification per section were analyzed to obtain the mean values. Percentage of IAS-AT infiltration was determined by calculating the ratio of adipose tissue area to total tissue area. To minimize the bias, the variables in the autopsy study were measured by two investigators who were blinded to the background information related to the enrolled patients (Miyoshi M and Abe I).

### Electron microscopy

To visualize the sectional ultrastructure of IAS-AT and the atrial myocardium, the IAS specimens were used for SEM. They were deparaffinized with xylene, hydrated, fixed again in Karnovsky’s fixative, and immersed in 2N NaOH at 37 °C for 3 h to expose myofibrils. Specimens were placed in 1% osmium tetroxide, 1% tannic acid, and 1% osmium tetroxide for 1 h each, dehydrated in ethanol solutions of ascending concentrations, and then dried using the tert-butyl alcohol freeze-drying method. The specimens were coated with gold and examined at 25 or 15 kV on a scanning electron microscope (S-4800; Hitachi High-Technologies, Tokyo, Japan).

### Statistical analyses

Continuous data were evaluated for normality with the Shapiro–Wilk test. Normally distributed continuous data were expressed as the mean ± standard deviation. To test for equal variance, either the F-test or the Levene-test was conducted. If the results were not significant, Student's *t*-test was used for two groups or One-way ANOVA was used for three groups. If the One-way ANOVA was significant, the Tukey–Kramer post hoc test was employed to identify significant differences between groups. If the F-test or Levene-test was significant, Welch's correction was applied. Multiple logistic regression analysis was used to compare several explanatory variables. Variables included IAS-AT volume, BMI, age, EpAT volume, age, gender, CHADS_2_ score, estimated glomerular filtration rate (eGFR), LVEF, and LAD, which were chosen a priori based on clinical relevance. A value of *p* < 0.05 was considered statistically significant.

All statistical tests were performed with JMP v.11 software (SAS, Cary, NC, USA) using Windows 10 (Microsoft™, Redmond, WA, USA).

## Supplementary Information

Supplementary Information.

## Data Availability

The datasets used and/or analyzed during the current study are deidentified and included in this published article (and its Supplementary Information files).

## Abbreviations

AF: Atrial fibrillation
BMI: Body mass index
EpAT: Epicardial adipose tissue
IAS: Interatrial septum
IAS-AT: Interatrial septum adipose tissue
TEE: Transthoracic echocardiography
TTE: Transthoracic echocardiography
CT: Computed tomography
LA: Left atrium
RA: Right atrium
PAF: Paroxysmal AF
PerAF: Persistent AF
LAD: Left atrial diameter
LVEF: Left ventricular ejection fraction
SAT: Subcutaneous adipose tissue
SEM: Scanning electron microscopy
HE: Hematoxylin–eosin
