# Supplementary material for: Association between interatrial septum adiposity and atrial fibrillation: transesophageal echocardiography imaging and autopsy study
Source: Sci Rep. 2023 Jun 17;13:9828. doi: 10.1038/s41598-023-36677-1 (PMC10276811; doi:10.1038/s41598-023-36677-1)
Supplement: Supplementary file 1 — Supplementary Information. [file 41598_2023_36677_MOESM1_ESM.docx]

**Supplemental materials**

**TITLE: Association between interatrial septum adiposity and atrial fibrillation:**

**Transesophageal Echocardiography Imaging and Autopsy study**

**AUTHORS:** Miho Miyoshi, Ichitaro Abe, Nozomi Kodama, Yinge Zhan, Shintaro Kira, Yumi Ishii, Taisuke Harada, Masayuki Takano, Masaki Takahashi, Hiroki Sato, Katsunori Tawara, Hidekazu Kondo, Akira Fukui, Tomoko Fukuda, Hidefumi Akioka, Tetsuji Shinohara, Yasushi Teshima, Kunio Yufu, Mikiko Nakagawa, Tsutomu Daa, Tatsuo Shimada, Naohiko Takahashi

**Supplemental Methods**

**Analysis bias**

To minimize the bias, the computed tomography (CT) and transesophageal echocardiography (TEE) imaging variables were measured by two investigators who were blinded to the background information related to the enrolled patients. Interobserver comparisons showed good agreement for interatrial septum (IAS)-thickness (r = 0.92) and epicardial adipose tissue (EpAT) volume (r = 0.90). Interobserver variability for IAS-thickness was 2.1%. Interobserver variability for EpAT volume was 5.4%. Intraobserver variability was determined by repeated assessment of IAS-thickness and EpAT volume by one investigator at different times. Intraobserver variability of IAS-thickness was 6.9% (r = 0.97). Intraobserver variability of EpAT volume was 7.2% (r = 0.99).

**Myocardial splitting by interatrial septum adipose tissue (IAS-AT)**

The interatrial septum adipose tissue (IAS-AT) infiltrated into the IAS myocardium in autopsy samples, as if adipose tissue split the myocardium. This characteristic finding was designated as “myocardial splitting by IAS-AT”. Myocardial splitting by IAS-AT was defined as clear myocardial compartmentalization by IAS-AT, which was observed at the border between atrial myocardium and IAS-AT. As a result of myocardial splitting by IAS-AT, several pieces of island-like myocardium were observed. Three random images, including the border between atrial myocardium and IAS-AT, at 40× magnification per section were analyzed in order to manually count the number of island-like myocardium pieces in each of all 10 autopsies to obtain the mean values. To minimize the bias, the variables in the autopsy study were measured by two investigators who were blinded to the background information related to the enrolled patients.

**Supplemental Figures**

**
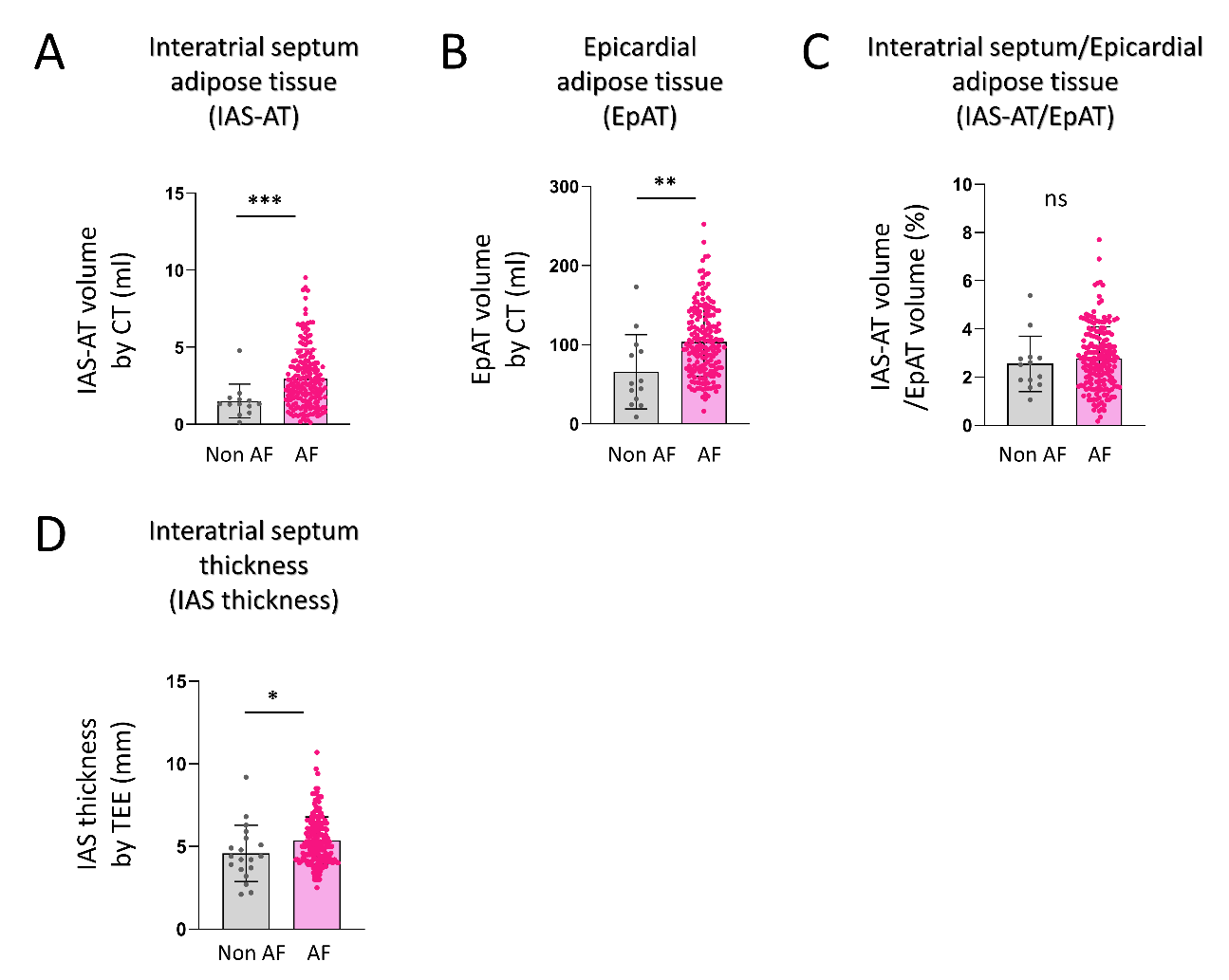
**

**Supplemental Figure S1. Comparison of interatrial septum adipose tissue (IAS-AT) volume among patients without AF (Non AF) and those with AF (AF). A.** Interatrial septum adipose tissue (IAS-AT) volume assessed by CT. Data are presented as means ± SD. *** *p* < 0.001, by Welch’s correction. *n* = 19 for Non AF, *n* = 184 for AF. **B.** Epicardial adipose tissue (EpAT) volume assessed by CT. Data are presented as means ± SD. ** *p* < 0.01, by Student’s *t*-test. *n* = 19 for Non AF, *n* = 184 for AF. **C.** IAS-AT volume/EpAT volume assessed by CT. Data are presented as means ± SD. ns, not significant by Student’s *t*-test. *n* = 19 for Non AF, *n* = 184 for AF. **D.** Interatrial septum (IAS) thickness assessed by transesophageal echocardiography (TEE). Data are presented as the means ± SD. * *p* < 0.05, by Student’s *t*-test. *n* = 19 for Non AF, *n* = 184 for AF.


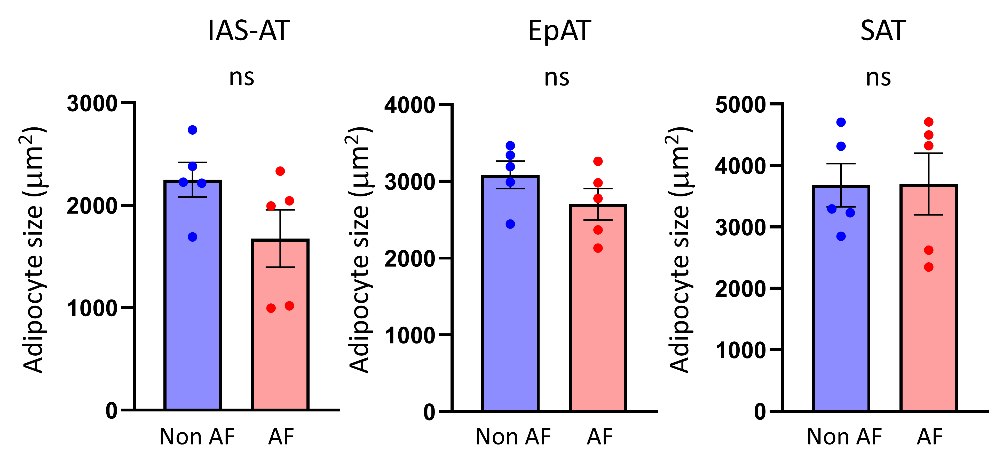


**Supplemental Figure S2. Adipocyte size in interatrial septum adipose tissue (IAS-AT), epicardial adipose tissue (EpAT), and subcutaneous adipose tissue (SAT) from Non AF and AF group.** Quantitative analysis of adipocyte size in IAS-AT, EpAT, and SAT from Non AF and AF patients. Data are presented as the means ± SD. ns; not significant by Student’s *t*-test. *n* = 5 for Non AF, *n* =5 for AF.

**
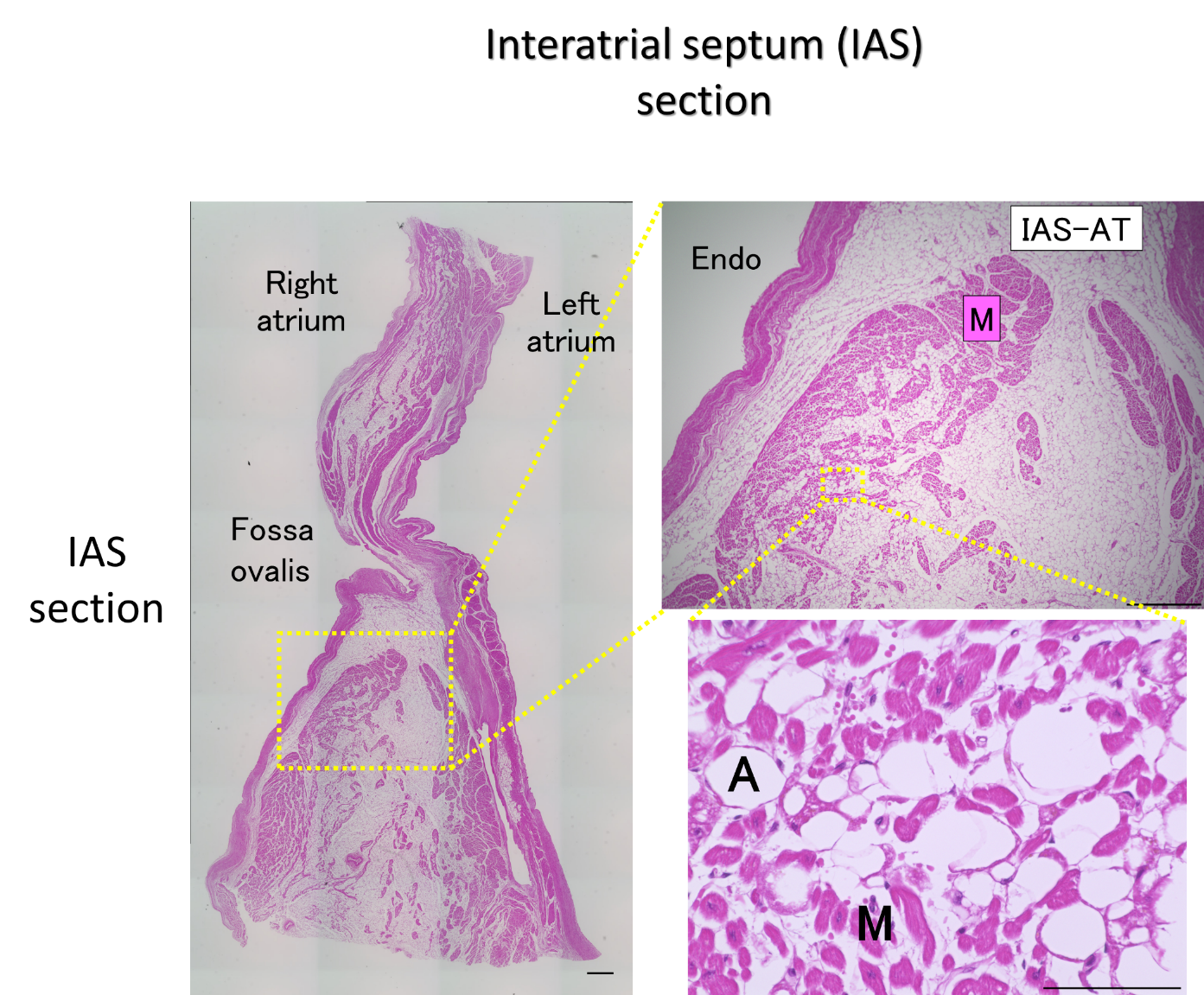
**

**Supplemental Figure S3. Myocardial splitting by interatrial septum adipose tissue (IAS-AT)**

Representative hematoxylin and eosin (HE) staining of interatrial septum (IAS) sections in patients with AF. Adipose tissue was commonly observed in human IAS samples. Adipocyte infiltration splits atrial cardiomyocytes. IAS-AT, interatrial septum adipose tissue; A, adipocyte; M, myocardium. Scale bar: 1 mm (left), 1 mm (upper), 100 μm (bottom).

**
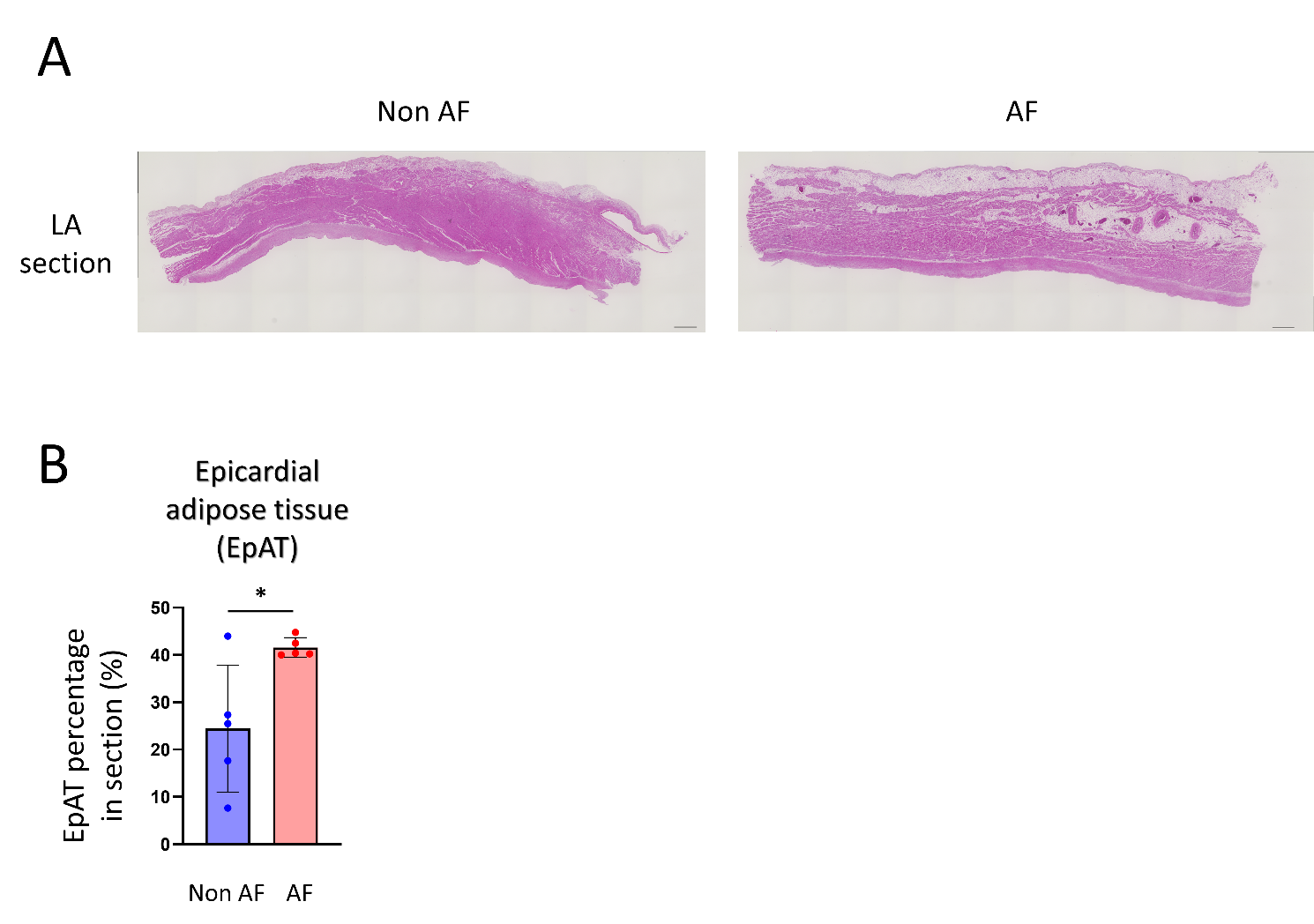
**

**Supplemental Figure S4. Histological analysis of human left atrium**

1. Representative images of left atrial (LA) sections in patients with and without AF.

**B.** Corresponding quantitative analysis of epicardial adipose tissue (EpAT) percentage in LA section. Scale bar: 1 mm, * *p* < 0.05, by Welch’s correction. *n* = 5 for Non AF, *n* =5 for AF


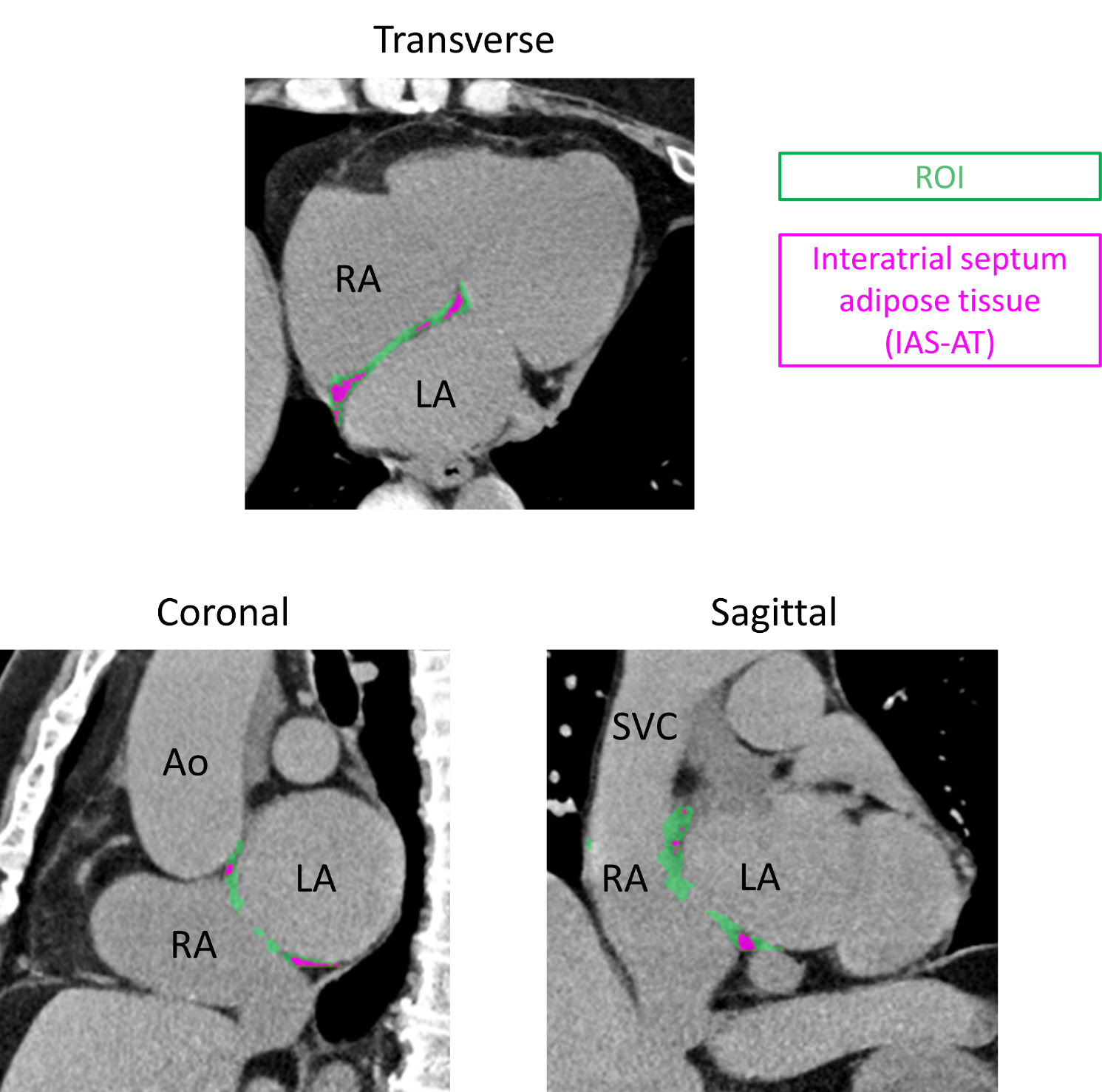


**Supplemental Figure S5. Quantification of interatrial septum adipose tissue (IAS-AT) by CT.**

Representative measurement images of interatrial septum adipose tissue (IAS-AT). Green highlighted portion was recognized as interatrial septum. Pink highlighted portion was recognized as IAS-AT. Ao, aorta; LA, left atrium; RA, right atrium; SVC, superior vena cava.

**
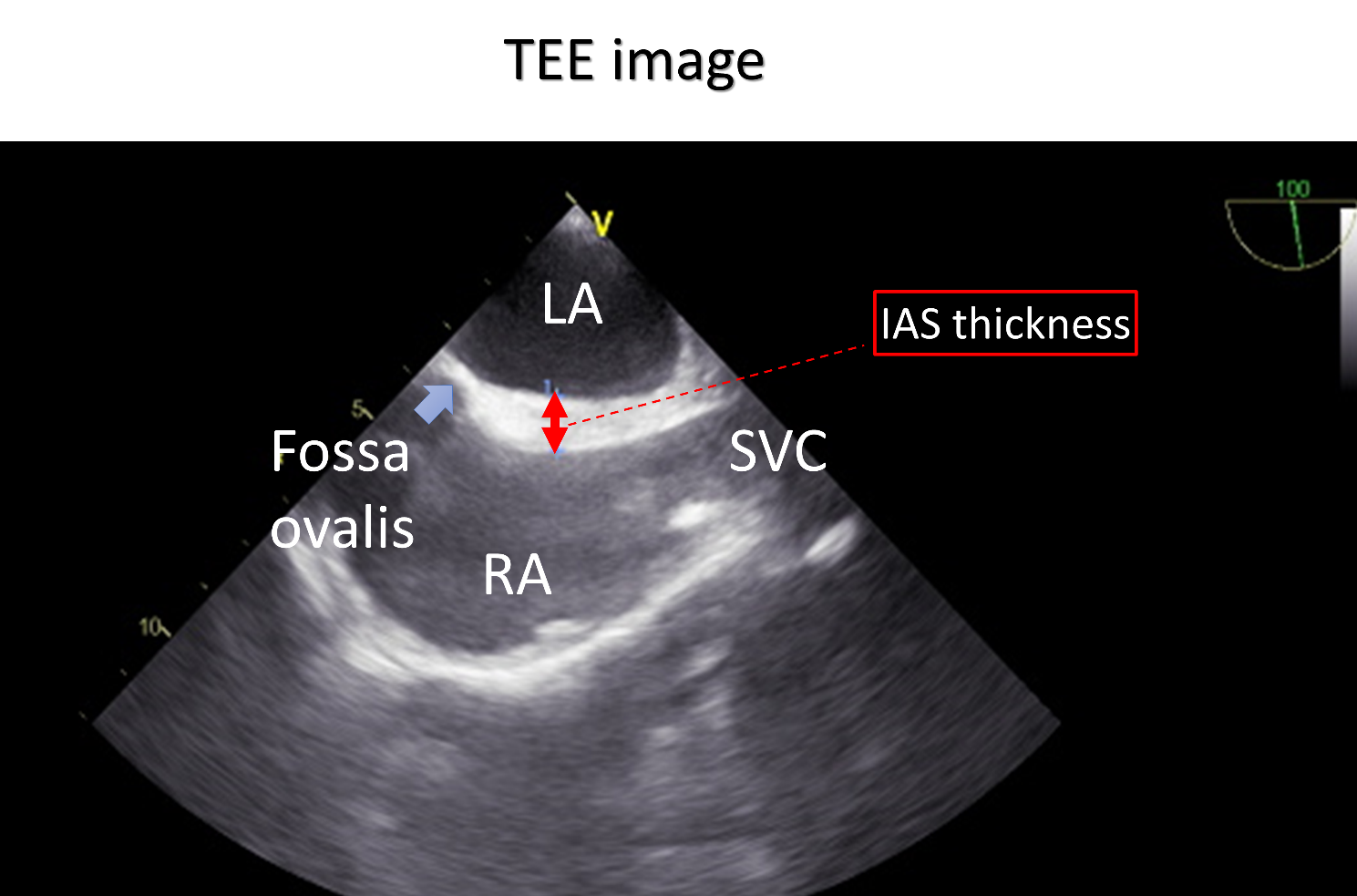
**

**Supplemental Figure S6. Quantification of interatrial septum (IAS) thickness by transesophageal echocardiography (TEE).**

Representative measurement image of interatrial septum (IAS) thickness. LA, left atrium; RA, right atrium; SVC, superior vena cava.

**Supplemental Table S1.**

Univariate analysis of correlation of IAS-AT volume assessed by CT

|  | *p* value | *r* |
| --- | --- | --- |
| BMI (kg/m₂) | < 0.01 | 0.48 |
| EpAT volume by CT (ml) | < 0.01 | 0.66 |
| LAD (mm) | < 0.01 | 0.28 |

IAS-AT = interatrial septum adipose tissue; CT = computed tomography; BMI = body mass index; EpAT = epicardial adipose tissue; LAD = left atrial dimension.

**Supplemental Table S2.**

Univariate analysis of correlation of IAS-AT volume assessed by CT in PAF group

|  | *p* value | *r* |
| --- | --- | --- |
| BMI (kg/m₂) | < 0.01 | 0.56 |
| EpAT volume by CT (ml) | < 0.01 | 0.68 |
| LAD (mm) | < 0.01 | 0.34 |

Univariate analysis of correlation of IAS-AT volume assessed by CT in PerAF group

|  | *p* value | *r* |
| --- | --- | --- |
| BMI (kg/m₂) | < 0.01 | 0.40 |
| EpAT volume by CT (ml) | < 0.01 | 0.64 |
| LAD (mm) | 0.12 | 0.17 |

IAS-AT = interatrial septum adipose tissue; CT = computed tomography; PAF = paroxysmal atrial fibrillation; PerAF = persistent atrial fibrillation; BMI = body mass index; EpAT = epicardial adipose tissue; LAD = left atrial dimension.

**Supplemental Table S3.**

Univariate analysis of correlation of IAS Thickness assessed by TEE

|  | *p* value | *r* |
| --- | --- | --- |
| BMI (kg/m₂) | < 0.01 | 0.41 |
| EpAT volume by CT (ml) | < 0.01 | 0.47 |
| IAS-AT volume by CT (ml) | < 0.01 | 0.52 |

IAS = interatrial septum; TEE = transesophageal echocardiography; BMI = body mass index; EpAT = epicardial adipose tissue; CT = computed tomography; IAS-AT = interatrial septum adipose tissue.

**Supplemental Table S4.**

Univariate analysis of correlation of IAS Thickness assessed by TEE in PAF group

|  | *p* value | *r* |
| --- | --- | --- |
| BMI (kg/m₂) | < 0.01 | 0.41 |
| EpAT volume by CT (ml) | < 0.01 | 0.47 |
| IAS-AT volume by CT (ml) | < 0.01 | 0.51 |

Univariate analysis of correlation of IAS Thickness assessed by TEE in PerAF group

|  | *p* value | *r* |
| --- | --- | --- |
| BMI (kg/m₂) | < 0.01 | 0.34 |
| EpAT volume by CT (ml) | < 0.01 | 0.47 |
| IAS-AT volume by CT (ml) | < 0.01 | 0.54 |

IAS = interatrial septum; TEE = transesophageal echocardiography; PAF = paroxysmal atrial fibrillation; PerAF = persistent atrial fibrillation; BMI = body mass index; EpAT = epicardial adipose tissue; CT = computed tomography; IAS-AT = interatrial septum adipose tissue.

**Supplemental Table S5.**

Patient characteristics of 203 patients.

|  | All patients (*n*=203) | Non-AF (*n*=19) | AF (*n*=184) |
| --- | --- | --- | --- |
| Age (years) | 67.3±11.8 | 54.1±22.9 | 68.7±8.9 ** |
| Sex |  |  |  |
| Male, n (%) | 140 (69) | 7(37) | 133 (72) * |
| Female, n (%) | 63 (31) | 12(63) | 51 (27) * |
| BMI (kg/m^2^) | 24.4±3.27 | 23.4±4.6 | 24.5±3.1 |
| CHADS_2_ score | 1.6±1.2 | 1.68±1.52 | 1.63±1.16 |
| HT, n (%) | 118 (58) | 5(26) | 113 (61) ** |
| DM, n (%) | 45 (22) | 4(21) | 41 (22) |
| HF, n (%) | 39 (19) | 3(16) | 36 (20) |
| Medication |  |  |  |
| ACEI or ARB, n (%) | 81 (40) | 4(21) | 77 (42) |
| *β*-blocker, n (%) | 88 (43) | 3(16) | 85 (46) * |
| Statin, n (%) | 69 (34) | 5(26) | 64 (35) |
| Amiodarone, n (%) | 52 (26) | 0(0) | 52 (28) * |
| Labo data |  |  |  |
| Hb (g/dl) | 13.7±1.8 | 12.1±1.9 | 13.8±1.7 ** |
| Cr (mg/dl) | 1.1±1.1 | 0.73±0.2 | 1.10±1.18 |
| eGFR (ml/min/1.73 m^2^) | 62.7±19.3 | 73.4±18.4 | 61.7±19.1 * |
| pro BNP (pg/ml) | 894.3±2864.4 | 349.2±305.9 | 918.8±2925.3 |
| HbA1c (%) | 5.9±0.6 | 6.1±0.7 | 5.9±0.6 |
| LDL-C (mg/dl) | 105.3±31.4 | 91.6±27.5 | 106.5±31.4 |
| HDL-C (mg/dl) | 57.4±15.4 | 58.9±23.5 | 57.3±14.4 |
| TG (mg/dl) | 114.2±68.7 | 91.7±59.4 | 116.3±69.1 |
| Echo data |  |  |  |
| LAD (mm) | 40.3±6.0 | 34.7±6.3 | 40.9±5.6 ** |
| LVDd (mm) | 48.3±5.7 | 46.3±7.2 | 48.5±5.5 |
| LVDs (mm) | 31.7±6.3 | 28.5±5.0 | 31.9±6.3 * |
| LVEF (%) | 63.0±11.3 | 66.2±5.6 | 62.7±11.6 |
| E/e' | 12.5±5.2 | 10.2±4.2 | 12.7±5.3 |

Data are given as mean±SD or n (%). * *p* < 0.05, ** *p* < 0.01, vs Non-AF.

AF = atrial fibrillation; BMI = body mass index; HT = hypertension; DM = diabetes mellitus; HF = heart failure; ACEI/ARB = angiotensin converting enzyme inhibitors/angiotensin II receptor blocker; Hb = hemoglobin; Cr = creatinine; eGFR = estimated glomerular filtration rate; pro BNP = pro brain natriuretic peptide; HbA1c = glycated hemoglobin; LDL-C = low density lipoprotein cholesterol; HDL-C = high density lipoprotein cholesterol; TG = triglyceride; LAD = left atrial diameter; LVDd = left ventricular end-diastolic diameter; LVDs = left ventricular end-systolic diameter; LVEF = left ventricular ejection fraction.

**Supplemental Table S6.**

Patient characteristics of 10 autopsies

|  | **Case**  **No.** | **Age** | **Sex** | **Cause of death** | **BMI**  **(kg/m^2^)** |
| --- | --- | --- | --- | --- | --- |
| **Non-AF group** | 1 | 72 | Female | Aortic aneurysm | 22.0 |
|  | 2 | 82 | Male | Amyloidosis | 27.6 |
|  | 3 | 76 | Female | Liver cirrhosis | 20.1 |
|  | 4 | 68 | Male | Sepsis | 22.1 |
|  | 5 | 63 | Female | Liver cirrhosis | 20.0 |
| **AF group** | 6 | 72 | Female | Mixed connective tissue disease | 20.0 |
|  | 7 | 76 | Male | Aortic aneurysm | 32.0 |
|  | 8 | 62 | Female | Liver cancer | 24.9 |
|  | 9 | 75 | Male | Amyloidosis | 18.7 |
|  | 10 | 84 | Male | Sepsis | 24.9 |

AF = atrial fibrillation; BMI = body mass index.

**Supplemental Table S7.**

|  | Non-AF group | AF group |
| --- | --- | --- |
| Age | 72.2±7.3 | 73.8±7.9 |
| Female/Male | 3/2 | 2/3 |
| BMI (kg/m^2^) | 22.3±3.1 | 24.1±5.2 |

AF = atrial fibrillation; BMI = body mass index.
